# Comparing the effectiveness of Family Support for Health Action (FAM-ACT) with traditional community health worker-led interventions to improve adult diabetes management and outcomes: study protocol for a randomized controlled trial

**DOI:** 10.1186/s13063-022-06764-1

**Published:** 2022-10-03

**Authors:** Denise J. Deverts, Michele Heisler, Edith C. Kieffer, Gretchen A. Piatt, Felix Valbuena, Jonathan G. Yabes, Claudia Guajardo, Deliana Ilarraza-Montalvo, Gloria Palmisano, Glory Koerbel, Ann-Marie Rosland

**Affiliations:** 1 University of Pittsburgh School of Medicine, Pittsburgh, PA USA; 2 University of Michigan, Ann Arbor, MI USA; 3 Community Health and Social Services Center, Inc., Detroit, MI USA

**Keywords:** Type 2 diabetes, Community health workers, Self-management interventions, Dyadic intervention, Social support, Family support, Peer support, Latino/a, Community-based participatory research

## Abstract

**Background:**

Diabetes self-management education and support (DSMES) programs have struggled to deliver sustainable, effective support for adults with diabetes (AWDs) to improve self-management behaviors, achieve glycemic goals, and reduce risk for complications. One largely untapped resource for this support is AWDs’ social networks. Fifty to 75% of AWDs have an unpaid family member or friend (“support person”) who provides ongoing help with diabetes management. However, DSMES interventions to date lack structured and effective approaches to directly engage support persons in AWDs’ diabetes management.

**Methods:**

This parallel arm randomized trial is designed to determine the effectiveness of Family Support for Health Action (FAM-ACT), a novel community health worker (CHW)-delivered program focused on educating and supporting patients with type 2 diabetes (T2D) and their support persons (SPs), relative to an established, CHW-delivered, individual patient-focused DSMES and care management (I-DSMES) intervention. Both interventions were developed using a community-based participatory research (CBPR) approach.

The study will be conducted in partnership with an urban Federally Qualified Health Center (FQHC) serving a low-income, Latino/a community, with target enrollment of 268 dyads consisting of an FQHC patient with T2D with high HbA1c and an SP. Patient-SP dyads will be randomized to receive FAM-ACT or I-DSMES over 6 months.

The primary outcome is change in patient HbA1c from baseline to 6 months. Secondary patient outcomes include 12-month change in HbA1c, changes in patient blood pressure, diabetes self-management behaviors, diabetes distress, patient activation, diabetes self-efficacy, and perceptions of and satisfaction with SP support for diabetes. Secondary SP outcomes include self-efficacy for helping the patient with diabetes management and SP distress about the patient’s diabetes. We also will assess the effect of the COVID-19 pandemic on patient’s ability to manage diabetes.

**Discussion:**

This study will inform scalable, evidence-based approaches that leverage family support to help AWDs improve and sustain self-management strategies that underpin optimal management of multiple diabetes complication risk factors. The protocol is designed for and evaluated with a low-income and predominantly Latino/a community, which may increase applicability to other similar communities. The COVID-19 pandemic presented several challenges to study protocol and intervention delivery; modifications made to address these challenges are described.

**Trial registration:**

ClinicalTrials.gov NCT03812614. Registered on 18 January 2019.

**Supplementary Information:**

The online version contains supplementary material available at 10.1186/s13063-022-06764-1.

## Administrative information

Note: the numbers in curly brackets in this protocol refer to SPIRIT checklist item numbers. The order of the items has been modified to group similar items (see http://www.equator-network.org/reporting-guidelines/spirit-2013-statement-defining-standard-protocol-items-for-clinical-trials/).

**Table:** 

Title {1}	Comparing the effectiveness of the Family Support for Health Action (FAM-ACT) with traditional Community Health Worker-led interventions to improve adult diabetes management and outcomes: study protocol for a randomized controlled trial
Trial registration {2a and 2b}.	ClinicalTrials.gov, NCT03812614, 18 January 2019 https://clinicaltrials.gov/
Protocol version {3}	Version 2.3, 27 June 2022
Funding {4}	National Institute of Diabetes and Digestive and Kidney Diseases, R01DK116733
Author details {5a}	University of Pittsburgh School of Medicine, Division of General Internal Medicine Community Health and Social Services Center, Inc University of Michigan School of Social Work University of Michigan School of Medicine University of Michigan School of Public Health VA Pittsburgh Center for Health Equity Research and Promotion
Name and contact information for the trial sponsor {5b}	National Institute of Diabetes and Digestive and Kidney Diseases 9000 Rockville Pike Bethesda, MD 20892
Role of sponsor {5c}	Funders had no role in study design, analysis, interpretation, report writing, or the decision to submit the report for publication.

## Introduction

### Background and rationale {6a}

The prevalence of type 2 diabetes (T2D) is growing [1, 2]. Many adults with diabetes (AWDs) have uncontrolled risk factors and thus are at high risk for diabetes complications [3, 4]. People with low socio-economic status and those in some racial and ethnic groups are at particularly high risk for developing T2D and, among those who already have been diagnosed with diabetes, to have worse glycemic control and higher rates of diabetes complications [5–9].

Risk for complications from T2D can be reduced through behavioral strategies that are key to managing glucose, blood pressure, and cholesterol levels. Optimally, these strategies are applied across several self-management tasks, then sustained and adapted to new diabetes management challenges over time. Diabetes self-management education and support (DSMES) programs are designed to help people with diabetes identify and overcome challenges to self-management and empower them to engage in behaviors that can help them more successfully manage diabetes. In previous studies, however, gains achieved from standard DSMES typically are not sustained beyond the program [10]. Moreover, programs that provide professional support for sustaining diabetes self-management improvements achieved in the short-term (e.g., 6 months) are costly and participants can struggle to maintain diabetes management gains after the program ends [11].

One important source of additional diabetes self-management support that remains largely underutilized by DSMES programs is patients’ family and other close social contacts. Fifty to 75% of AWDs report involving family members or friends in their health care [11–15]. These “family supporters” or “support persons” assist AWDs in activities directly related to successful diabetes management, including medication management and adherence, tracking home glucose and blood pressure measurements, maintaining a healthy eating plan, and engaging in physical activity [11, 12, 16, 17]. Family supporters also assist AWDs with day-to-day challenges of diabetes self-management, such as helping address medication side effects [14]. Family supporters typically are spouses (50–60%), with most other supporters being family members who do not live with the patient (e.g., adult children) [12, 18, 19].

Adults with T2D and other chronic health conditions that are complicated by low health literacy, multiple comorbidities, or comorbid depression involve family supporters in their care more often than those without these concurrent challenges [15–17, 19, 20]. Family supporters also are involved in patients’ interactions with the healthcare system. Over half of adults with chronic health conditions are regularly accompanied by family into the exam room for primary care visits, and 25% have had a supporter talk on the phone with their healthcare providers in the last year [14, 15, 17, 21]. Findings from several observational studies demonstrate that among persons with chronic health conditions, family support is associated with better health outcomes, especially among patients whose conditions are complicated by the need for complex self-management regimens. Specific examples include better glycemic control and lower mortality among AWDs and lower rates of recurrent cardiac events and hospitalizations among persons with cardiac disease and heart failure [22–27]. Among AWDs, higher levels of family support also are associated with less diabetes distress [28, 29]. There is evidence to suggest that the observed association of social support with chronic disease outcomes may be due to improved patient self-management behaviors [30–32]. Despite these promising observational findings, it remains unclear how best to maximize the benefits of patients’ existing social support in interventions designed to improve diabetes outcomes.

Previous studies with interventions that simply provide family members of AWDs with general information about diabetes or invite family members to attend standard, exclusively patient-focused programs with the AWD are not effective [33–36].﻿ There is increasing evidence that family supporters may be more effective in supporting AWDs if they take on the role of effectively supporting instrumental diabetes tasks, such as taking medications or home glucose monitoring [31, 37, 38]. Just as critical as the type of self-management support family supporters provide, is *how* they go about providing that support. Self Determination Theory suggests that, to be effective, support should prioritize patient autonomy and increase patients’ confidence in their own ability to enact targeted self-management behaviors [39–41]. Thus, the most effective dyadic or family interventions may be those that emphasize family supporters’ provision of “autonomy support.” Autonomy support is defined as the degree to which healthcare providers and supportive others empower patients by understanding patients’ priorities and needs, acknowledge patients’ feelings, provide meaningful self-management choices, and avoid controlling the patients’ behavior [39]. Autonomy and empowerment-based interventions delivered by healthcare professionals have been shown to improve diabetes health outcomes more than control conditions [40–44]. Recent observational studies show strong associations between autonomy support from family and AWDs’ self-management behaviors and glycemic control [45, 46]. A pilot study training family members in autonomy supportive communication had a significant effect on lowering sodium intake among adults with heart failure over 3 months [47, 48]. However, autonomy support training for family members to date has not been incorporated into comprehensive family support interventions.

In addition to focusing on family support for patient autonomy and self-efficacy, interventions aimed at integrating family support roles into AWDs’ daily self-management may be even more effective if they can be adapted to community and cultural norms using a community-partnered research approach. For AWDs from several racial and ethnic communities, including supportive family and friends in DSMES programs may be especially effective [34, 49]. Several studies have found that Latino/a and African-American AWDs desire more family involvement in diabetes self-management [50], and family members of AWDs in these groups express a willingness to support their loved ones with diabetes self-management tasks [14]. Latino/a adults in particular have high family involvement in health care [51–53]. Latino/a family members often take on unique roles such as adapting traditional foods, translating and navigating health care encounters, and encouraging mutually appealing physical activities [54]. Health management often is considered to be a family responsibility [55–57].

This study builds on the long-standing Racial & Ethnic Approaches to Community Health (REACH) Detroit community-academic partnership that has addressed health disparities in Latino/a and Black adults with T2D, using robust Community Based Participatory Research (CBPR) processes [53, 58–60]. In studies completed prior to this current study, the REACH Detroit Partnership developed and tested the efficacy of culturally tailored DSMES curricula and community health worker (CHW) interventions for diabetes that were effective in improving glycemic control and health behaviors over 6 months compared to control groups among mostly Latino/a and Black AWDs [58–61].

In summary, family supporters play important roles in diabetes management for many AWDs, and social support has been linked to better diabetes health outcomes. Due to their established trust and accessibility, family members may be able to take on unique support roles, particularly in families in Latino/a communities, but previous dyadic studies that give supporters patient-focused diabetes information have not improved adults’ diabetes management. Promising approaches to engaging family supporters of AWDs focus on helping supporters identify their own roles in supporting diabetes-specific tasks that meet AWDs’ goals, coaching supporters in the use of positive and autonomy-supportive communication to support AWDs’ goals, and tailoring program content to community and cultural context through community partnerships.

This study seeks to test Family Support for Health Action (FAM-ACT), a CHW-delivered program that integrates these promising approaches to improve and sustain healthy diabetes management and outcomes. Specifically, FAM-ACT will (1) coach family supporters in regular discussions about AWDs’ diabetes progress and goals that use empathetic and autonomy-supportive communication, (2) coach family supporters in practical roles that support diabetes-specific tasks tailored to AWDs’ personal goals, (3) leverage family support in the setting of other types of social support for AWDs (support from other AWDs and their family members and CHWs), and (4) be developed and implemented in culturally concordant ways, in partnership with the community participating in the program.

### Objectives {7}

The objective of this study is to determine the effectiveness of FAM-ACT, a novel program focused on educating and supporting both AWDs and the family and friends who support them, relative to an individual patient-focused DSME and care management (I-DSMES) program. Both are community health worker (CHW)-led DSMES interventions.

The study objectives are to:

1. Determine the effectiveness of FAM-ACT, compared to I-DSMES, in improving AWDs’ diabetes-related health outcomes
2. Determine the effectiveness of FAM-ACT, compared to I-DSMES, in improving ADWs’ health behaviors and perceived support
3. Examine theoretical-model-driven moderators and mediators of FAM-ACT participation on patient outcomes

### Trial design {8}

**Fig. 1:**
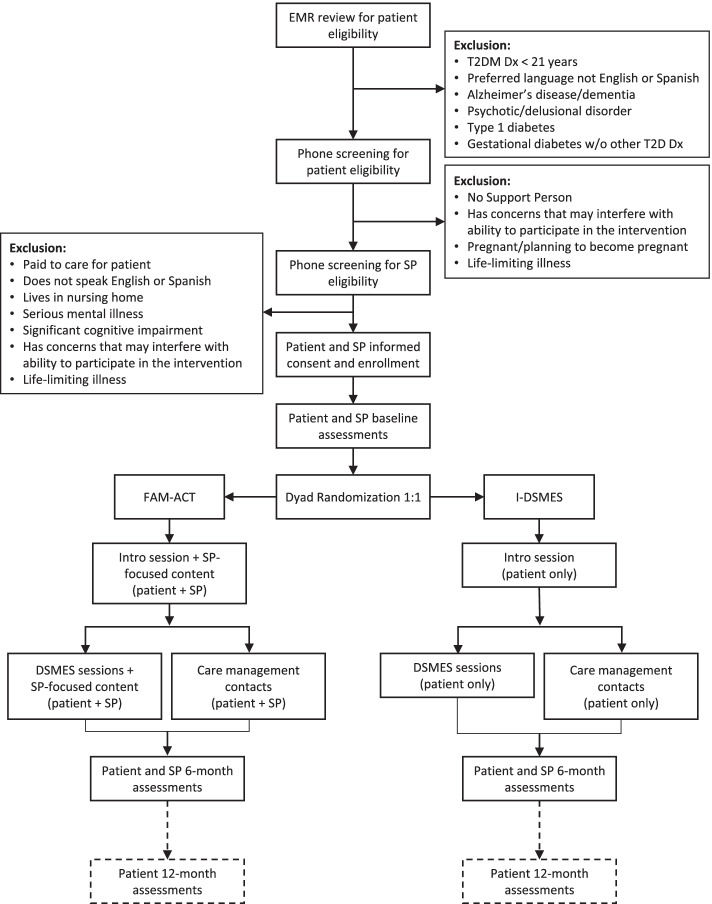
Participant flow through the protocol

The trial protocol was developed using CBPR approaches based on active academic and community partnership in every stage of study design. The study will be a single-site parallel arm randomized controlled superiority trial with dyads consisting of an adult with type 2 diabetes (“patient”) and a selected family supporter (“support person”). Dyads will be randomized 1:1 to either the FAM-ACT program or a traditional individual patient-focused I-DSMES program for 6 months. Assessments of both members of the dyad will be conducted at approximately 6 and 12 months following enrollment. Figure 1 provides a graphic representation of the study protocol.

The intervention initially had been designed to span 12 months (“original protocol”). This original protocol was used for the first 77 patient-support person dyads who enrolled in the study prior to March 2020, when the COVID-19 pandemic reached the USA and limits were placed on the conduct of in-person research activities. In response to the pandemic, several modifications were made to the protocol—including reducing the length of the intervention to 6 months—both to ensure the safety of study participants and research staff as well as to compensate for a temporary pause in enrollment due to pandemic-related restrictions on recruitment activities (“adapted protocol”). The adapted protocol was put into effect in February 2021, when recruitment activities were permitted to restart, and will remain in effect throughout the duration of the study. Differences between the original and adapted protocols will be highlighted in subsequent sections.

## Methods: participants, interventions, and outcomes

### Study setting {9}

The intervention site will be an urban, community-based US Federally Qualified Health Center (FQHC) in Detroit, MI, that serves a clientele that is majority Latino/a and Spanish-speaking. All patient participants will be established users of the FQHC’s healthcare services at the time of enrollment. Two-thirds of the FQHC’s patients are uninsured, and 24% have Medicaid insurance.

### Eligibility criteria {10}

#### Study participants

Study participants will be dyads comprised of patients and their enrolled support person (SP). Potentially eligible patients will be identified via review of FQHC patient electronic medical records (EMR) or FQHC clinician referral and provided a study information letter. Interested patients then will be screened by study staff by phone or in-person to verify whether they meet inclusion criteria.

##### Inclusion criteria

Adult FQHC patients must meet the following inclusion criteria at the time of screening:

- Is at least 21 years of age but no more than 75 years of age
- Has a diagnosis of type 2 diabetes mellitus in the FQHC EMR Problem List (ICD10 codes E08.xx, E09.xx, E11.xx, E13.xx, O24.1x, O24.3x, O24.8x, O24.9x)
- Has poor glycemic control, defined as HbA1c ≥ 7.5%, per the most recent test performed within the previous 3 months (the original protocol used HbA1c ≥ 8%; see the “Adaptations for COVID-19 pandemic” section)
- Plans to continue using the FQHC for health care throughout the following 12 months
- Is able to identify an adult family member or friend who is involved in their health care and is willing to be contacted about participating in the study with the patient

##### Exclusion criteria

Potential patient participants will be excluded from the study if any of the following criteria apply:

- Originally diagnosed with diabetes before the age of 21 years
- Has a diagnosis of type 1 diabetes mellitus in the FQHC EMR Problem List (ICD10 codes E10.xx, O24.0x)
- Has a diagnosis of gestational diabetes (ICD10 codes 024.4x) without any other diabetes diagnoses
- Preferred language is neither English nor Spanish
- Has a diagnosis (active or prior) of schizophrenia or other psychotic/delusional disorder in FQHC EMR Problem List (ICD10 codes F20.xx, F21.xx, F22.xx, F25.xx, F28.xx, F29.xx)
- Has a diagnosis (active or prior) of Alzheimer’s disease or dementia in FQHC EMR Problem List (ICD10 codes F01.50, F01.51, F02.80, F02.81, F03.90, F03.91, G23.1, G30.0, G30.1, G30.8, G30.9, G31.01, G31.09, G31.83)
- Has a life-limiting severe illness (e.g., chronic obstructive pulmonary disease requiring oxygen)
- Is pregnant or planning to become pregnant within the next 12 months
- Has other concerns that may significantly interfere with their ability to participate in the intervention (ongoing health issues, personal events, etc.)

Following eligibility screening, eligible and interested patients will be asked to identify a SP who might be willing to participate with them in the study. SPs will be screened by study staff in person or by phone using a separate set of screening questions.

Patient-nominated SPs must meet the following inclusion criteria at the time of screening:

- At least 21 years of age
- Able to attend intervention sessions in person or remotely via online videoconferencing if invited

Potential SP participants will be excluded from the study if any of the following criteria apply:

- Receives pay for caring for the patient
- Does not speak English or Spanish fluently
- Lives in a nursing home or other long-term care facility
- Has self-reported serious mental illness (e.g., schizophrenia)
- Has significant cognitive impairment (Alzheimer’s disease or dementia)
- Has concerns that may significantly interfere with their ability to participate in the intervention (ongoing health issues, personal events, etc.)
- Has a life-limiting severe illness (e.g., chronic obstructive pulmonary disease requiring oxygen)

#### Interventionists

##### Qualifications

Both the FAM-ACT and I-DSMES interventions will be delivered by FQHC-employed CHWs. Qualifications include a high school diploma or GED, fluency in Spanish and English, and familiarity with the local community.

##### Training

Qualified CHWs will receive CHW training and certification from the Michigan Community Health Worker Alliance (MiCHWA) and be trained to provide American Diabetes Association (ADA) Standards-Compliant Diabetes Self-Management Education, consisting of 20 h of coursework that is based on the skills and knowledge defined for Diabetes Paraprofessional Level 1. CHWs will then be trained in study protocols and intervention delivery by the PI and other study investigators and the experienced, certified CHW manager.

#### Adaptations for the COVID-19 pandemic

##### Original protocol

When initially conceived, the study planned to examine 5-year diabetes-specific cardiac risk (UK Prospective Diabetes Study [UKPDS] score) as a secondary outcome. For this reason, adults with diabetes would be eligible to participate in the study if (a) their most recent HbA1c is ≥ 8.0% and/or (b) has poor blood pressure control, defined as systolic blood pressure (SBP) > 150 mm Hg, recorded in the FQHC EMR at least twice in the past 6 months, with the most recent SBP being > 150 mm Hg.

##### Adapted protocol

Because the UKPDS score was dropped as an outcome (see the “Secondary outcomes” section), poor blood pressure control no longer is being used as an inclusion criterion, thus requiring all patient participants to qualify based on their HbA1c. Eliminating the blood pressure criterion effectively reduces the size of our eligible patient pool. To compensate for this limitation, the HbA1c criterion has been lowered to ≥ 7.5%.

### Who will take informed consent? {26a}

Informed consent from patients and their SPs will be obtained by trained study research assistants (RAs) who are fluent in both Spanish and English.

### Additional consent provisions for collection and use of participant data and biological specimens {26b}

Patient participants will be asked to consent to up to 3 study-conducted HbA1c, blood pressure, and weight assessments, and the use of diabetes-relevant EMR data from the time period of 12 months prior to enrollment to 12 months after the patient’s active participation in the study has ended. Patient participants are also asked to consent to FQHC staff discussing diabetes-related medical information with their enrolled SP during their active study participation period.

## Interventions

### Explanation for the choice of comparators {6b}

The effectiveness of the novel dyadic FAM-ACT intervention will be compared to a “gold-standard” ADA-guideline-compliant DSME curriculum that focuses on individual patients in combination with care management calls from a CHW. Previous research comparing standard DSMES to a usual care controls has shown a strong effect of DSMES on glycemic control over a 3- to 6-month time period, with continuing but diminishing effects for up to 24 months [62]. The FAM-ACT intervention builds upon the DSMES curriculum framework. Thus, every patient will receive evidence-based DSMES content as part of their intervention, as well as periodic care management contacts. This design will make it possible to distinguish the amount and type of “value added” by including structured SP participation and SP-focused content to standard DSME, as well as involving the SP in patients’ care management.

### Intervention description {11a}

#### FAM-ACT intervention

##### Overview

The FAM-ACT intervention will include (1) an introductory session that includes a patient and SP needs assessment and initial education about dyadic goal-setting and autonomy-supportive communication, (2) 6 group DSME sessions with additional SP role-focused information and discussion, and (3) dyad-focused care management contacts biweekly for 6 months. All intervention components have been designed to be usable by SPs who live in or outside of the patient’s household and will be offered in both Spanish and English. Patient-facing materials to be used or distributed to participants during the introductory and DSME sessions (e.g., patient handbook, informational hand-outs) have been written to accommodate individuals at low reading levels. All participant materials were created in English, with Spanish-language versions having been professionally translated from the English.

To develop the FAM-ACT intervention, 8 experienced, local CHWs participated in 4 focus group sessions to discuss their experiences with family participation in CHW-delivered chronic disease interventions. CHWs recommended emphasizing the potential for family support for diabetes management to decrease family distress about the patient’s health and help the patient remain healthy so they can participate in family activities. CHWs also assisted in adapting a draft intervention curriculum and protocols to the perceived needs of local community patients, ensuring that all participant materials are accessible to those with low literacy levels and available both in English and in Spanish tailored to the dialect of the local community.

##### Introductory (“Intro”) session

Once enrolled, patient-SP dyads will be invited to attend an initial in-person 60–90-min introductory session with a CHW. During this session, the CHW will review the patient’s diabetes complication risk profile (most recent HbA1c, blood pressure, cholesterol results, and smoking status) and current diabetes medication regimen with the patient and their SP. The CHW will then provide initial education to the dyad about productive and positive ways to work on diabetes goals together, including the use of autonomy-supportive communication. Finally, the CHW will provide education and guidance in setting an initial SP-enhanced “SMART” (specific, measurable, achievable, relevant, and time-specific) health goal, including a SP role *chosen by the patient* to support their goal.

##### SP-enhanced DSME sessions

Both patients and SPs will be encouraged to attend 6 group DSME sessions delivered by CHWs, although either member of the dyad may attend without the other if necessary. Six topical sessions will be delivered in an ongoing rotation, once every 2 weeks. Each session will last approximately 1.5–2 h and will use the ADA-approved *Conversation Maps*® to help guide dyads through the session material. Each session will be enhanced with a discussion of progress toward SPs increasing their involvement in the patient’s diabetes management, and skills SPs can use to address the diabetes management topics covered in the session (see Table 1). In addition to the support-focused topics listed in Table 1, all SP-enhanced sessions include discussions about positive communication techniques and patient-SP weekly talks about diabetes.

**Table 1:** Core DSME session topics and FAM-ACT program support person-focused material

Session	I-DSMES and FAM-ACT^a^	FAM-ACT only
1	• Patient’s experience and understanding of diabetes • Emotional aspects of diabetes • Information people with diabetes should know • Managing diabetes with healthy eating, physical activity, and medication adherence • Support networks	• Support for increasing physical activity • Ways to help patient understand and address mood changes • Possible stressors for the support person
2	• Patient’s understanding of diabetes • Feelings about food • Basic concepts about food and nutrients found in foods • The 5 major food groups • Making healthy food choices	• Support for healthy eating
3	• Portion sizes • Timing and frequency of meals • Strategies for healthy eating • Food challenges and planning	• Support for food planning • Support for challenging food situations
4	• The basics of blood glucose • Blood glucose targets • Glucose monitoring, knowing I1c • Factors that affect blood glucose levels • Managing high and low sugars	• How to recognize high and low sugars in the patient • Ways to help the patient prevent low sugars • Ways to help the patient treat low sugars • Support for glucose monitoring • Ways to help the patient manage sick days
5	• Review of previous sessions • Keeping blood glucose on target • Signs and symptoms of low and high blood glucose • Long-term diabetes complications • Checking for diabetes complications • ABCs of diabetes: A1c, blood pressure, and cholesterol	• Support for obtaining diabetes screening tests • Support for foot care • Support for recognizing health emergencies
6	• Oral medications and insulin • Medication adherence • Physical activity	*Support for patient when they are* • Having trouble remembering to take their medication • Out of medication • Not sure how to take their medication as prescribed • Worried about how much their medication costs • Worried about side effects • Worried they are taking too many pills for different conditions • Unable to pick up their medication from the pharmacy

^a^ All sessions also include a “goal-setting” component

##### Biweekly dyadic care management

Throughout the 6-month intervention, the CHW will reach out regularly to both the patient and the SP to conduct care management contacts by phone or video chat. During these calls, the CHW will review the patient’s progress on the action plan the patient and SP made during the previous contact. If the patient is satisfied with their progress, then the CHW will encourage them to work together with their SP to create a new plan to reach the next step toward the patient’s goals. If the patient is not satisfied with their progress, the patient, SP (if present on the call), and CHW will discuss the barriers that prevented the patient from making progress toward their goals and work with them on identifying ways they can work together to adapt the action plan to overcome these barriers. The CHW also will provide guidance for SPs on what they can do to help the patient when problem issues are detected, while reinforcing key principles of patient empowerment and autonomy-supportive communication.

#### I-DSMES intervention

##### Overview

The I-DSMES intervention will include (1) an introductory session that includes a patient needs assessment, (2) 6 group DSME sessions, and (3) patient-focused care management contacts biweekly for 6 months. Similar to FAM-ACT, all components of the I-DSMES intervention will be offered in both Spanish and English.

##### Introductory (“Intro”) session

Once enrolled, patients only will be invited to attend an initial in-person 60-min introductory session with a CHW. As with FAM-ACT, the CHW will review the patient’s diabetes complications risk profile and current diabetes medication regimen and then follow with an introduction to “SMART” goal-setting. Patients randomized to I-DSMES will not be provided with the additional information about SP roles and communication.

##### DSME sessions

Patients assigned to the I-DSMES intervention will be encouraged to attend the same 6 topical diabetes education sessions described above for FAM-ACT, but will not be invited to stay for the presentation of the supporter-focused content. SPs of patients in the I-DSMES intervention may attend the standard group sessions if they choose to do so. However, CHWs will not reach out directly to SPs of patients who have been assigned to I-DSMES to facilitate attendance.

##### Biweekly care management

Throughout the 6-month intervention, the CHWs will reach out regularly to the patient to conduct care management by phone or video chat. During these calls, the CHW will review the patient’s progress on the action plan the patient made during the previous contact. If the patient is satisfied with their progress, then the CHW will encourage them to create a new plan to reach the next step toward their goals. If the patient is not satisfied with their progress, the CHW will discuss the barriers that prevented the patient from making progress toward their goals and work with them on identifying ways they can change their action plan to overcome these barriers.

### Adaptations for the COVID-19 pandemic

#### Changes to the length of the intervention protocol

##### Original protocol

Both the FAM-ACT and I-DSMES interventions originally were designed to last 12 months, with the introductory and DSMES sessions completed during the first 6 months of the protocol, and care management contacts continuing throughout the subsequent 6 months. Most study contacts occurred in the first 6 months of the 12-month period.

##### Adapted protocol

The protocol has been condensed to complete the intervention in 6 months. This step was taken to compensate for the inability to recruit new patients and SPs during the several-month pandemic-related pause in research study recruitment and the additional time needed to make substantial changes to the study and intervention approaches. The shorter protocol allows time for full sample recruitment, with time to complete the protocol, within the timeframe of study funding.

#### Changes to introductory and DSME session delivery

##### Original protocol

Intervention sessions originally were designed to be delivered in an in-person setting at the FQHC, with each session lasting between 90 min and 2 h. Conducting the group sessions in an in-person setting enables the CHW to use the ADA-approved DSMES *Conversation Maps*® and other visual aids to help guide participants through the session material. This hands-on approach is designed to encourage participant engagement with the educational material as well as provide participants with an opportunity to interact with other patients with diabetes. The face-to-face setting also facilitates communication and relationship-building between the participant and CHW.

##### Adapted protocol

To accommodate restrictions on in-person and group contact during the height of the pandemic, introductory sessions and group education sessions for both interventions have been redesigned to be offered on a virtual platform (Zoom). Several changes were made to both the structure and content of the DSME sessions to facilitate virtual delivery, including reducing the duration of the sessions to 45–60 min (to prevent “Zoom fatigue”), replacing the ADA-approved DSME *Conversation Maps*® with online PowerPoint slides, and creating revised facilitator guides that are better suited to virtual delivery of the DSMES intervention.

The study CHWs based the content of the presentations and facilitator guides on the *Conversation Map*® curriculum and incorporated engaging illustrations and other graphics to approximate the visual learning experience offered by the *Conversation Maps*®. To reduce the length of the education sessions without sacrificing essential content, CHWs will pose fewer discussion questions during the core and SP-enhanced DSMES sessions and eliminate redundancy by decreasing the number of examples being used to explain a given concept.

As safety protocols permit, participants will be offered a choice of in-person or virtual introductory sessions. All DSMES group sessions will be hybrid (virtual + in-person).

#### Changes to biweekly care management contacts

##### Original protocol

Care management contacts from the CHW originally were designed to be delivered predominantly by phone or video chat, but also in-person at the FQHC during clinical visits, or at the participant’s home if necessary.

##### Adapted protocol

To accommodate social distancing guidelines and participant and CHW health precautions, in-person care management contacts have been eliminated.

### Participant materials

All patients, and SPs who have been assigned to FAM-ACT, will receive a hardcopy study workbook and a set of printed information documents and worksheets (“handouts”). All materials have been written to be understandable at a 6th-grade reading level and are available in Spanish and English.

Two versions of the study workbook were created: one for use by patients and SPs assigned to FAM-ACT and another for patients assigned to I-DSMES. Both versions of the workbook contain general information about type 2 diabetes and diabetes self-management. The FAM-ACT workbook also contains information about the roles played by SPs in patients’ diabetes care. Table 2 lists the topics that are covered in both versions of the workbook, as well as information specific to the FAM-ACT version.

**Table 2:** Topics covered in participant workbooks

Topics covered in workbooks for both interventions	Topics covered in FAM-ACT workbook only
• Basic physiology of type 2 diabetes	• Positive communication techniques, including autonomy-supportive communication
• Diabetes complications	• Support for diabetes self-care activities
• Basic information about blood pressure and cholesterol	• Support of blood sugar monitoring and ways to help treat low blood sugar
• Smoking	• Support for taking medication
• Body mass index (BMI)	• Ways SPs can help when the patient is sick
• Glucose monitoring	• Support for preparing for healthcare provider visits and interpreting medical tests
• Goal-setting	• Support for caring for diabetes complications
	• SP support for patient goal-setting

#### Adaptations for the COVID-19 pandemic

##### Original protocol

Per the original procedure, the CHW was responsible for distributing workbooks and printed handouts. Workbooks were to be given to participants when they met with the CHW for the introductory session, and topical handouts were to be distributed during the relevant DSMES session.

##### Adapted protocol

To accommodate participants who attend sessions virtually, patients now receive all educational materials when they come to the clinic for their baseline assessment with the RA. If a patient assigned to the FAM-ACT intervention lives with their SP, they also are given the SP’s educational materials. If the SP does not live with the patient, the RA will send the SP their materials via postal mail.

### Intervention fidelity

Fidelity monitoring will be conducted by the principal investigator, co-investigators, and CHW manager and will be performed either synchronously (in-person or via Zoom) or asynchronously using an audio recording of the participant contact to be monitored. Monitored contacts will include those conducted in both English and Spanish.

Monitors will use fidelity checklist forms that were created for this study to evaluate a specified number of each type of structured participant contact (recruitment call, informed consent process, introductory session, survey assessments, and DSMES group sessions). Checklists contain items relating to both the content of the specific contact to be evaluated as well as the interpersonal or group facilitation skills of the CHW or RA who was involved in the contact. Following the evaluation, the monitor will review the completed checklist form (Additional file 1_Fidelity-Checklist) with the involved CHW or RA and provide feedback as needed to maintain the fidelity of the intervention and assessments. The monitoring schedule for each type of contact is described in Additional file 2_Fidelity-Monitoring-Schedule.

### Criteria for discontinuing or modifying allocated interventions {11b}

#### Disabling health events

Study participants (patients or SPs) may be removed from active participation in the intervention if they experience a significantly disabling health event that would make continued participation difficult. However, if the participant elects not to withdraw completely, they will be retained in the study and contacted for planned study assessments, with the data being included in intent-to-treat (ITT) study analyses.

#### Significant separation from SP

If a patient participant experiences a significant separation in relationship with their SP (e.g., divorce from a spouse), the SP may be removed from active participation in the intervention. However, if the patient elects not to completely end the SP’s participation, the SP will be retained in the study and contacted for planned study assessments.

#### Violent behavior and incarceration

Any participant (patient or SP) will be withdrawn from study participation if they (a) exhibit violent, threatening, or harassing behavior toward study or FQHC site staff or (b) experience incarceration or are placed on parole during their participation period.

#### Pregnancy

Patients will be withdrawn from the study if they become pregnant while participating in the study and will be advised to seek medical advice regarding diabetes management during pregnancy with their usual primary care provider as soon as possible.

#### Strategies to improve adherence to interventions {11c}

Several strategies will be used to facilitate attendance, such as offering the sessions at rotating times and arranging transportation to the FQHC when needed. Despite these accommodations, it is expected that some participants will miss some sessions. In this case, the CHW will deliver a condensed version of the missed content during planned care management contacts. This approach was shown to be effective in this research groups’ previously published CHW diabetes interventions [58–61].

### Relevant concomitant care permitted or prohibited during the trial {11d}

Patients will be encouraged to continue with their usual medical care while participating in the study. Enrolled SPs in dyads assigned to the I-DSMES arm will not be prohibited from attending study intervention sessions or other medical care visits with the enrolled patient if they choose of their own accord to do so.

### Provisions for post-trial care {30}

There is no anticipated harm or need for post-trial care or compensation.

## Outcomes {12}

### Primary outcome

The study’s primary outcome is change in patient hemoglobin A1c (HbA1c) from baseline to 6 months post-baseline.

### Secondary and other prespecified outcomes

**Fig. 2:**
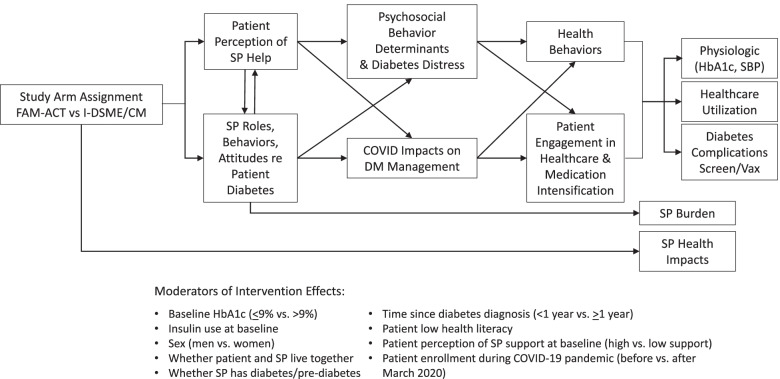
Conceptual model

Pre-specified secondary patient outcomes include baseline to 12-month change in HbA1c, 6-month and 12-month change in blood pressure, 6-month and 12-month change in diabetes distress, and 6-month change in specific behavioral determinants, self-management behaviors, and perceived support metrics (see Table 3 for detailed list). Prespecified secondary SP outcomes include change in diabetes distress, as measured using the Problem Areas in Diabetes (PAID)-5 for Family Members Scale [63] and efficacy for supporting the patient’s diabetes management using the Self-Efficacy for Managing Chronic Diseases Scale [64] (adapted for support persons). Baseline to 12-month change all secondary patient outcomes listed above (save for HbA1c, blood pressure and diabetes distress) will be included as *other pre-specified outcomes*. Additional exploratory outcomes assessed may include changes over time in other domains indicated in the study conceptual model (Fig. 2).

**Table 3:** Pre-specified patient secondary outcomes^a^

Measure	Concept category	Instrument(s)	Baseline	6 months	12 months^b^
Physiologic measures^c^					
** Glycemic control**	Clinical	Hemoglobin A1c (%)	X	**X**	X
Blood pressure control	Clinical	Systolic blood pressure (mmHg)	X	X	X
Survey measures					
Diabetes distress	Psychological behavior determinants	Problem Areas in Diabetes (PAID)-5 for People with Diabetes [63]	X	X	X
Self-reported healthy eating	Self-management behaviors	Summary of Diabetes Self-Care Activities Measure (SDSCA) [65]	X	X	*X*
Self-reported physical activity	Self-management behaviors	Summary of Diabetes Self-Care Activities Measure (SDSCA)	X	X	*X*
Self-reported medication adherence	Self-management behaviors	Summary of Diabetes Self-Care Activities Measure (SDSCA)	X	X	*X*
Patient activation	Psychological behavior determinants	Patient Activation Measure (PAM-13) [66]	X	X	*X*
Diabetes self-efficacy	Psychological behavior determinants	Self-Efficacy for Managing Chronic Diseases Scale [64]	X	X	*X*
Patient overall satisfaction with SP support for diabetes	Patient perception of SP help	Patient overall satisfaction with SP support items created for CO-IMPACT Study [67]^,d^	X	X	*X*
Patient perceived supportive vs. non-supportive SP behaviors					
Autonomy support (“supportive behaviors”)	Patient perception of SP help	Important Other Climate Questionnaire (IOCQ) [68]	X	X	*X*
Non-supportive behaviors	Patient perception of SP help	3 items created for this study^e^	X	X	*X*
Impact of COVID on ability to manage diabetes	COVID-19 impact	Single item created for study^f^		X	*X*

Bold, change in measure from baseline to 6 months is the primary outcome; italics, change in measure from baseline to 12 months is an *other pre-specified outcome*

^a^ Baseline to 6-month change in all of the measures listed in the table (save for 6-month change in HbA1c), as well as baseline to 12-month change in HbA1c, blood pressure, and diabetes distress, are included as secondary outcomes. Changes from baseline to 12 months in all other measures are included as other pre-specified outcomes

^b^ Per the COVID-adapted protocol, 12-month assessments will be completed only if the study timeline permits

^c^ Physiologic data will be collected by study research assistants either directly during a scheduled study assessment, or using patient EMR data (including an extended period of 12 months pre to 18 months post baseline)

^d^ Two items assessing patient’s satisfaction with the support they receive from their SP and whether they feel like they would be worse off without their SP’s help with their diabetes care

^e^ Non-supportive behaviors will be assessed with 3 items structured similarly to the IOCQ items addressing SP irritation, criticism, and argumentativeness

^f^ “In the last six months, how have the COVID pandemic or social distancing rules affected your ability to manage your diabetes?” (5-point scale, 1—much harder to 5—much easier)

### Adaptations for the COVID-19 pandemic

#### Primary outcome

##### Original protocol

The original primary outcome was change in patient HbA1c from baseline to 12 months post-baseline, reflecting the original intervention length of 12 months.

##### Adapted protocol

The primary outcome was revised to change in HbA1c from baseline to 6 months, reflecting the adapted intervention length of 6 months.

### Secondary outcomes

#### Original protocol

The original analysis plan included 6-month and 12-month change in the patient 5-year diabetes-specific cardiac risk (UKPDS score) as a secondary outcome. The calculation of the UKPDS score is based on HbA1c, systolic blood pressure, cholesterol levels, and smoking status.

#### Adapted protocol

Change in UKPDS score has been dropped as a secondary outcome because patient cholesterol levels no longer can be reliably measured without in-person assessment. A substantial number of patients from the first recruitment wave became eligible for their 6-month assessment during the time that restrictions on in-person research activities were in effect.

## Participant timeline {13}

**Fig. 3:**
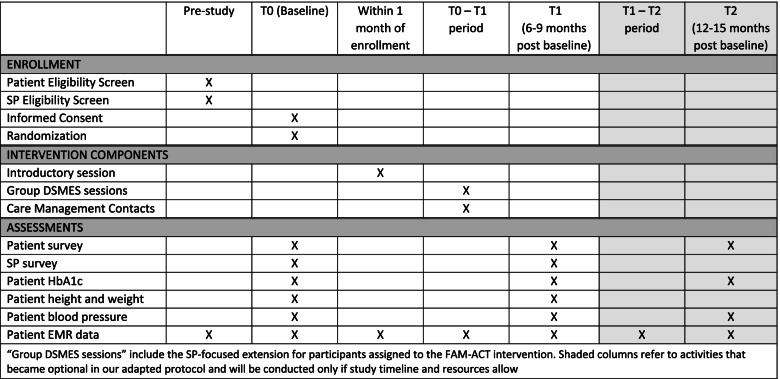
Schedule of standard protocol elements

Figure 3 illustrates the schedule of standard protocol elements. Participants will be assessed at baseline (T0) and at 6–9 months post-enrollment (T1). If time and resources allow, patient participants also may be assessed 12–15 months post-enrollment (T2).

## Sample size {14}

### Original protocol

The study has been powered to detect a clinically meaningful difference in HgbA1c at 12 months based on a linear mixed model with an intervention arm, linear time (T0, T1, and T2), and the intervention arm-by-time interaction. Because allocation to the study arm will be randomized, calculations were based on the assumption that the mean difference at baseline is zero and that the intervention effect is represented by the between-group difference in time slope (captured by the intervention arm-by-time interaction), or differences at follow-up time points. All calculations assumed at least 80% power and 2-sided tests at level *α*= 0.05 using the R package longpower [69]. The standard deviation of HbA1c at any given time point was estimated in a range based on findings from previous studies at the FQHC (SD range 1.65–2.05%), a previous study of peer support for diabetes at a similar study site (SD 1.75%), and an estimate from a general published trial (SD 1.5%) [58, 60, 70, 71]. The correlation between time points, as measured by the intra-class correlation coefficient (ICC), ranged from 0.50 to 0.7 based on the same diabetes trial data from the study site. To detect a 12-month mean difference of 0.6% in HgbA1c, the total sample size required is 214. This sample size was based on the assumption of an HgbA1c standard deviation (SD = 1.75%) and ICC (ICC = 0.6) within the range of potential values reported in the literature. If these estimates were conservative (e.g., if true SD = 1.5% and true ICC = 0.7), this sample size achieves at least 80% power to detect a mean HbA1c difference as small as 0.4%. Anticipating 20% attrition based on the rate observed in prior RCTs conducted at the FQHC, target enrollment will be 268 dyads (134 dyads per intervention arm).

### Adapted protocol

The study was not resized after modifying the primary outcome timepoint from 12 to 6 months. The study team determined that detecting a 0.6% difference in HbA1c within 6 months, rather than 12 months, is reasonable because preliminary data used in the original power analyses were based on a 6-month duration and the impact of the intervention is anticipated to be greatest within 6 months.

## Recruitment {15}

### Patient recruitment

#### Provider referral/EMR

Patients identified by FQHC provider referral or site EMR data pull will be mailed a study recruitment letter. Recipients will have the opportunity to opt-out of the study by calling the study phone number. If they do not opt out within 3 days of the recruitment letter being sent, study staff will call the patient to continue the recruitment and screening process.

#### Recruitment flyer

Recruitment flyers will be posted in the FQHC clinic, distributed at community health-related events and posted on the FQHC Facebook, Twitter, and Instagram sites. Interested patients will contact the study staff via the phone number provided on the flyer.

#### Warm handoff

If FQHC clinical providers or study staff identify potentially eligible patients during clinic visits, study staff will meet with the patient in-person at the clinic to describe the study if the patient is agreeable.

### Support person (SP) recruitment

Eligible and interested patients will be asked to choose a family member or friend to participate with them in the study as their SP. Patient-selected SPs will be sent a recruitment letter and study information sheet, using the contact information provided by the patient. If the SP does not opt out within 3 days of the recruitment letter being sent, a study RA will call the SP to continue the recruitment and screening process.

## Assignment of interventions

### Allocation

#### Sequence generation {16a}

Participants will be allocated in a 1:1 ratio to either FAM-ACT or I-DSMES using a computer-generated randomization series concealed within an automated Web-based randomization system. The randomization will use a permuted block design with random block sizes of 2 and 4, stratified by baseline HbA1c (≤ 9% versus > 9%) and whether the patient and support person live together.

#### Concealment mechanism {16b}

A randomization function using the aforementioned algorithm will be programmed into the study database. This function will remain locked until the RA documents that patient informed consent has been obtained and the baseline assessment data have been entered.

#### Implementation {16c}

Following entry of all required information, the randomization function will be enabled, allowing the RA to obtain the code indicating whether the patient and their SP will be assigned to FAM-ACT or I-DSMES.

## Blinding

### Who will be blinded? {17a}

The PI, co-investigators, and data analysts will be blinded to the patient-SP dyad intervention assignment. The CHW and study staff cannot be blinded to allocation due to the nature of the intervention, but outcome assessors will be unaware of group allocation and statistical summaries for trial monitoring will contain no group identifiers. Unblinded data evaluation during the trial will be restricted to the data center conducting the analyses. Investigators will be unblinded and analyses will begin only after all data collection forms have been completed, data queries resolved, and data are locked for analysis.

### Procedure for unblinding if needed {17b}

Unblinding will be based entirely on the patient and/or SP safety and only under circumstances when knowledge of the dyad’s group assignment is essential to ensuring their safety.

## Data collection and management

### Plans for assessment and collection of outcomes {18a}

Patient biological assessments and patient and SP survey assessments will be conducted at baseline (T0) and at 6 to 9 months post-baseline (T1/ “6-month assessment”). Patients also will be assessed at 12 to 15 months post-baseline (T2/ “12-month assessment”) if the study timeline and resources permit the additional assessment.

### Patient measures

#### Patient HbA1c

Patient HbA1c will be measured using a portable fingerstick sample analyzer (Bayer DCA 2000® + , Bayer Diagnostics, USA), which is accurate and has a coefficient of variation < 5% as required by the National Diabetes Data Group [72]. If HbA1c cannot be obtained at a dedicated study visit, HbA1c values in the clinic EMR, which are collected using the same fingerstick analyzer, will be recorded in place of the study assessment.

#### Systolic blood pressure

Patient blood pressure will be measured by a trained staff member using an oscillometric upper arm blood pressure monitor (Omron® 3 Series, Omron Healthcare, Inc., USA) and following American Heart Association guidelines [73]. Two readings will be taken 2–5 min apart, and the average of these 2 readings will be used as the measure of blood pressure. As with HbA1c, if blood pressure data cannot be obtained at a dedicated study visit, blood pressure values recorded in the clinic EMR closest to the target assessment date, but within 2 weeks before and after, will be used.

#### Health behaviors and psychosocial behavior determinants

Health behaviors and psychosocial behavior determinants will be measured via self-report survey instruments. Surveys will be read aloud by a study RA in the participant’s preferred language either in-person or by phone, and responses will be recorded by the RA. Select patient survey measures are presented in Additional file 3_Additional-Patient-&-SP-Measures (Supplementary Table 1). All Spanish-language surveys were professionally translated.

### Support person (SP) measures

SP data will be measured via self-report survey instruments, administered in-person or by phone by study RAs using the same method as patient surveys above, at baseline and 6–9 months post-baseline. Assessed constructs include psychological behavior determinants and SP roles in, behaviors relating to, and attitudes toward the patient’s diabetes management. Select survey measures are presented in Additional file 3 (Supplementary Table 2). As with the patient surveys, Spanish-language SP surveys were professionally translated.

### Adaptations for the COVID-19 pandemic

#### HbA1c measurement

##### Original protocol

Per the original protocol, patients were to have their HbA1c assessed at 3 time points: baseline, 5 to 7 months post-baseline, and 11 to 13 months post-baseline. All assessments were to be conducted by study RAs using the fingerstick method described above.

##### Adapted protocol

To accommodate pandemic-related restrictions on in-person research activities, 2 additional HbA1c assessment methods have been incorporated into the protocol.

*Clinic measures*: Tests unable to be obtained in-person during the assessment window will be recorded from the patient’s electronic medical record (EMR) rather than obtained via a study staff-conducted assessment. When appropriate, CHWs will contact patients to ask if they can schedule a time for their provider-ordered clinical assessment or labs on a day that would ensure the test(s) also occurs within the study assessment window.

*Home testing*: Patients may be offered the option to self-test their HbA1c using a fingerstick device (e.g., A1cNow® Self Check, pts Diagnostics) sent to their home. Patients will be provided with illustrated, easy-to- understand instructions for using the device, as well as live assistance (by phone or video chat) from study RAs.

#### Survey assessments

##### Original protocol

As part of the original protocol, SPs were to be assessed by survey at 2 timepoints: baseline and 11–13 months post-baseline.

##### Adapted protocol

Because the duration of the study protocol was decreased from 12 to 6 months, SPs now will be surveyed at baseline and 6–9 months post-baseline.

### Plans to promote participant retention to complete follow-up {18b}

A combination of effective methods will be used to maximize participant retention, including pre-DSMES session reminder calling, follow-up calls for missed sessions, updating contact information at each visit, collecting backup friend/family contact information, distributing materials with study phone numbers (e.g., refrigerator magnets), and providing pre-addressed stamped post-cards to send to the study team in the event contact information changes. Using these strategies, prior studies with this population had > 80% retention in CHW interventions over 12 months [58–61].

### Data management {19}

Data will be entered electronically via a password-protected Web-based data entry system created using ASP.NET programming. Assessment data will be entered by a study RA in real time as the data are being collected. Data will be stored off-site at a University data coordinating center using MS SQL Server and will be backed up daily.

During data entry, a number of strategies will be employed to ensure data quality: use of standard methods of data collection and recording already specified in study protocols, careful programming of the data management system, detailed documentation of computer operations and data editing procedures, and regular meetings with project staff to review any changes in procedure. The data coordinating center will verify all data, program out-of-range data checks into data entry fields, and evaluate the full data process within and across forms.

### Confidentiality {27}

Patient and SP confidentiality will be maintained to the highest extent possible throughout all phases of the study. Each patient-SP dyad will be assigned a unique case number, with this number being the only link between the participant and their research records. Consent forms and other identifying information will be stored separately from participants’ research records and maintained in a secure location. Audio recordings for fidelity assessment will be labeled with a study identification number only and will be destroyed immediately following internal fidelity review by authorized study staff.

All study personnel will complete training in maintaining patient confidentiality and will sign a written statement indicating that they will preserve the confidentiality of participant research records as a condition of their employment.

Any breach of confidentiality will be reported immediately to the PI and to the Institutional Review Board (IRB). Likewise, any complaints or concerns expressed to the study staff by participants, providers, or other persons affected by this study will be reported immediately to the PI and the IRB.

### Plans for collection, laboratory evaluation, and storage of biological specimens for genetic or molecular analysis in this trial/future use {33}

All laboratory evaluations will be conducted using fingerstick blood samples. There will be no short- or long-term storage of participant blood or other biological specimens.

## Statistical methods

### Statistical methods for primary and secondary outcomes {20a}

The primary analysis will be intent-to-treat (ITT), with the primary hypothesis to be tested being change in HbA1c from baseline to 6 months differs between FAM-ACT and I-DSMES.

Changes in HbA1c over time initially will be examined graphically. Then, main analyses will be conducted using linear mixed-effects models that include repeated measures at baseline, 6 months, and 12 months, with random patient intercepts and random time slopes. To evaluate the effect of the intervention over time, we will include time in the model plus an interaction term for time-by-intervention arm. Models with time as a categorical rather than continuous variable will be explored, with be best-fitting model (based on Akaike’s Information and Schwarz’s Bayesian Criteria) being used. The primary hypotheses (change from baseline to 6 months) will be tested using linear contrasts. Treatment effect estimates will be presented along with 95% confidence intervals. The model will be adjusted for design variables (baseline HbA1c and whether the patient and SP live together), baseline insulin use, and age. If baseline HbA1c and insulin use are highly correlated, HbA1c will be prioritized in the primary model, with adjustment for insulin use in sensitivity analyses.

Secondary health behavior and social support outcomes will be analyzed in a similar manner. Generalized linear mixed models with logit link will be used for categorical and ordinal outcome measures. Intervention effects at 6 months and 12 months will be estimated and tested for significance using linear contrasts.

For all analyses, the overall level of significance will be set to *α* = 0.05. Data analyses will be performed using SAS v9.4 (SAS Institute, Cary, NC) or the latest version of R. To achieve maximum power for the primary endpoint, and because analyses involving the secondary outcomes, mediation, and moderation are considered hypothesis generating, no adjustment for multiple testing will be used.

### Interim analyses {21b}

There are no planned interim analyses.

### Methods for additional analyses (e.g., subgroup analyses) {20b}

#### Exploratory mediators and moderators

Baseline participant characteristics are expected to vary, thus allowing for the examination of whether these variables moderate the effectiveness of the interventions. Prespecified subgroup analyses will be conducted to understand the intervention effect, and to identify subgroups of patients for whom the intervention was particularly beneficial and/or harmful. Pre-specified key moderators/subgroups are baseline HbA1c ≤ 9% vs. > 9%, patient uses insulin at baseline (Y/N), sex (man/woman), whether patient and support person live together (Y/N), whether support person has diabetes or pre-diabetes (Y/N), time since diabetes diagnosis (< 1 year vs. ≥ 1 year), patient low health literacy (Y/N), patient level of SP support at baseline (high vs low), and patient enrollment relative to the COVID pandemic start (before vs after March 2020).

Additional exploratory moderators and mediators of outcomes analysis will be selected for analyses based on the study conceptual model (Fig. 2).

Moderation of intervention effectiveness will be examined by including an interaction of the variable of interest with the group-by-time term in the analytic models. A significant interaction term would indicate moderation, and the moderated effect estimates will be reported from the interaction model. If significant moderation is found, estimates and confidence intervals of the intervention effect within each subgroup defined by the moderator will be reported.

Natural effect models based on a counterfactual framework will be applied to examine mediation effects [74, 75]. A 2-way decomposition of the total effect of the intervention on outcomes into an average direct effect and average indirect effect (i.e., other than through the mediator) will be adopted.

### Methods in analysis to handle protocol non-adherence and any statistical methods to handle missing data {20c}

The extent and reasons that data are missing will be described by study arm. Baseline patient characteristics will be compared between those who have complete outcome data and those who do not.

The primary analytic approach (mixed models) can validly handle missing-at-random data. However, as appropriate and consistent with the planned approach to handle intercurrent events (ICE), multiple imputations will be employed to conduct the ITT analyses.

In line with the ICH E9 R1 guidelines, potential study ICEs will be identified and a strategy that most aligns with the clinical question of interest will be selected for the primary analyses. Supplementary analyses will examine the impact of intervention in an adherent population by estimating the complier average causal effect (CACE).

### Plans to give access to the full protocol, participant level-data and statistical code {31c}

The full final study protocol will be made available online as supplementary material to the main outcome publication. Final research data will be shared in accordance with the most recent NIH guidelines (https://grants.nih.gov/grants/policy/data_sharing/), while being mindful that the confidentiality and privacy of participants in research must be protected at all times. All data sharing will follow institutional policies and local IRB rules, as well as local, state, and Federal laws and regulations including the HIPAA Privacy Rule. To protect the rights and privacy of individuals, any data that will be shared outside of the research team will be free of identifiers that would permit linkages to individual research participants and variables that could lead to deductive disclosure of the identity of individual subjects. De-identified data and statistical code used in published analyses will be made available upon written request to the principal investigator as described in a later section (see the “Availability of data and materials” section).

## Oversight and monitoring

### Composition of the coordinating center and trial steering committee {5d}

The PI, study coordinator, and CHW manager will meet biweekly during the planning phase to refine intervention materials, train CHWs, and plan for recruitment. Co-investigators will meet monthly and will review all key study materials and procedures. During the intervention phase, the PI and all study staff (study coordinator, CHW manager, CHWs, and RAs) will have biweekly cross-site staff meetings to review recruitment, reasons for declining/dropout, and protocol delivery. In addition, the PI, study coordinator, and CHW manager will meet weekly to address any study management issues. The CHW manager, CHWs, and RAs will meet at least weekly to monitor daily study activities and address urgent issues. The study monitor will attend all cross-site staff and co-investigator meetings. In the analysis phase, the PI, statistician, and data analyst will meet bimonthly, with input from other co-investigators at key points. Ad hoc analysis meetings will be scheduled as needed.

### Composition of the data monitoring committee, its role, and reporting structure {21a}

The funder and IRB have determined that the study is minimal risk, and thus, a formal data monitoring committee is not required. Instead, a detailed data monitoring plan will be followed that includes a single study data monitor.

### Adverse event reporting and harms {22}

Study RAs and CHWs will follow a clear protocol that includes immediate reporting of adverse events (AEs) and potential problems. Additionally, the informed consent document will provide contact information for both the study PI and IRB to facilitate self-report of adverse events. Appropriate entities (i.e., IRB, NIDDK) will be notified regarding all study-related serious adverse events (SAEs) within standard reporting guidelines.

All study-related SAEs will be categorized according to the NCI Common Terminology Criteria for Adverse Events (CTCAE). A designated safety monitor (the PI or site-PI) will be tasked with adjudicating all study-related SAEs with the aid of discharge summaries uploaded to the study’s Web-based data management system. A log detailing reported study-wide SAEs will be maintained, including those events that are study-related and those unrelated to study participation.

### Frequency and plans for auditing trial conduct {23}

Bi-monthly study team meetings, monthly data review, and periodic site visits by the PI (when safety protocols permit) will be conducted to assess data quality and ensure that IRB policies and procedures are being followed. This review will include ensuring that (1) all patients understand, agree to, and sign a written consent form prior to engaging in any research activities; (2) strict adherence is maintained to communication regarding the participants’ right to withdraw or refuse to answer questions; (3) staff maintain confidentiality by protecting hard-copy and electronic data collection forms and by avoiding all unauthorized conversations about individual patients; (4) consent forms and identifying information are kept separately from study-related information about patients’ sociodemographics, clinical characteristics, disease self-care, service use, and outcomes; (5) all identifying information is kept locked at all times and sensitive computer files are maintained on a secured server; (6) coding for ambiguous responses is handled in a way that is consistent and clear across data collectors and over time; and (7) participants are informed in writing how to contact the study PI, the study project coordinators (FQHC & University of Pittsburgh), and the relevant IRB office with any questions or concerns.

### Plans for communicating important protocol amendments to relevant parties (e.g., trial participants, ethical committees) {25}

Important protocol amendments will be submitted to the IRB for approval prior to incorporating them into the existing protocol. If approved changes to the protocol affect any procedures yet to be performed by current participants, then participants will be contacted by a member of the study staff who will explain the changes to the protocol. Patients who are agreeable to the amended protocol will be asked to sign a revised consent form that addresses the amended procedures. SPs will provide verbal consent to the revised consent script.

## Dissemination plans {31a}

Study results will be disseminated through multiple communication strategies including academic conferences and publications, professional societies such as healthcare provider and diabetes educator organizations, healthcare system operations leaders, community organizations, and patient advocacy groups focused on diabetes. Participants will be mailed a layperson summary of results from the trial after all main analyses have been completed.

## Discussion

The design of this study has evolved in response to the many challenges posed by the COVID-19 pandemic. Temporary restrictions on the conduct of human subjects research during the height of the pandemic affected not only the pragmatic aspects of study conduct, but also the daily life experiences of the participants and the partner community. To meet these challenges, several adaptations were made to study recruitment strategies (including patient eligibility); consent and assessment processes; intervention content, delivery mode, and length; and outcomes of focus and analysis as outlined above.

The most significant challenge was the adaptation of an in-person intervention for virtual delivery, which required considerable time and resources to accomplish while still following CBPR procedures. Study CHWs and investigators used an iterative process to adapt the educational content initially provided using the visually engaging and interactive *Conversation Map*® curriculum for presentation online. They also selectively pared down the material to reduce the overall length of the education sessions and thus spare participants from becoming fatigued from spending 1–2 h engaged in online group sessions. Technical challenges included the selection of an online platform that met several criteria (e.g., easy to use, designed for multi-device optimization, HIPAA-compliant, free-of-charge) as well as the need to create graphics-enhanced user instructions that are accessible at low reading levels and available in English and Spanish.

Despite these and other challenges, adaptation of the intervention for delivery in a virtual or hybrid format has been accepted by participants. Though having less opportunity for face-to-face interaction, the adapted intervention has the advantage of being easier for some participants to access. Specifically, participants no longer are obligated to travel to the FQHC to attend the DSMES sessions. This benefit may be especially important for this study population because access to reliable transportation can be difficult in the area served by the partner FQHC. The virtual option also allows for the possibility of long-distance support. Per the original protocol, SP eligibility was dependent upon the SP living in close enough proximity to the FQHC to be able to attend the DSMES sessions with the patient if invited. Without that restriction, patients now have the option of selecting any family member or friend to be their SP, regardless of that person’s physical location.

Originally, the study proposed to examine pre- to post-intervention change in both HbA1c and UKPDS risk score as main outcomes. Thus, the plan was to recruit adults with a diagnosis of T2D with high glycemic levels and/or high systolic blood pressure. Due to pandemic delays in recruiting and resulting uncertainty about the study’s power to detect multiple key outcomes, the decision was made to streamline both the patient eligibility criteria and main outcome analyses. Now, and moving forward, recruitment will be limited to adults with a diagnosis of T2D and high glycemic levels. Patients no longer will be recruited based on meeting the “high blood pressure” inclusion criterion alone. Likewise, change in HbA1c will be the only primary outcome examined.

Even though diabetes-specific cardiac risk no longer will be included as a primary outcome, the study intervention’s introductory and DSMES sessions will continue to address the importance of blood pressure management for reducing risk for diabetes complications [76]. CHWs will review recent blood pressures with patients and include the option for patients to set SMART goals related to blood pressure management. Due to the increased emotional stress linked to having diabetes during the COVID pandemic, the intervention session content that addresses the emotional aspects of diabetes also will be emphasized. Given the documented links between increased social support, improved blood pressure control, and decreased diabetes distress, whether change in blood pressure and diabetes distress levels differ between FAM-ACT and I-DSMES will be examined as prioritized but exploratory secondary analyses.

## Conclusion

Despite the challenges posed by the COVID-19 pandemic, this trial is well poised to be completed as per the adapted protocol. The intended outcome of the trial is to produce a feasible strategy for optimizing the support available to at-risk adults with T2D from their existing social networks. The study’s ultimate goal is to mobilize that enhanced support to increase patients’ ability to successfully manage diabetes. This study also will address gaps in knowledge about the impact of different types of social support on the health outcomes of adults with T2D. Specific questions will focus on general social support, diabetes-specific support, autonomy support, and supporter roles in diabetes management, and how changes in each of these types of support affect health behaviors and outcomes over time.

The use of CBPR approaches to research and inclusion of CHWs in the processes of intervention design and implementation assures valuable contributions from the family culture and lived experiences of the partner community. This approach facilitated the development and administration of a family support-focused intervention that is tailored to the needs of that community. The community-partnered approach also enhances meaningful engagement with participants and provides opportunities to learn authentic lessons that can make a true impact on diabetes management for similar communities.

## Trial status

Initial recruitment into the FAM-ACT study began in September 2019. Restrictions on human subjects research made in response to the COVID-19 pandemic resulted in the temporary suspension of study recruitment from March 2020 to February 2021. Prior to the pause, recruitment proceeded uninterrupted, yielding a total of 77 randomized patient-SP dyads by March 2020. Recruitment is ongoing at the time of manuscript submission, and to date, 189/268 patient-SP dyads have been randomized.

Due to pandemic-related delays, and additional costs to revise recruitment, intervention, and assessment approaches, the NIH approved a request for a funding supplement, with an anticipated recruitment completion date of December 2022.

## Supplementary Information

**Additional file 1. **Fidelity Checklists. FAM-ACT Fidelity Checklists. This file contains the checklists used by the PI, co-investigators and CHW manager assessing structured contacts between study staff and participants for fidelity to the protocol. There is one checklist for each type of contact.

**Additional file 2.** Fidelity-Monitoring-Schedule. Intervention component fidelity monitoring schedule. The table located in the file displays the fidelity monitoring schedules by study contact, staff member and participant type.

**Additional file 3.** Additional-Patient-&-SP-Measures. Table 1, Select patient survey measures; Table 2, Select support person survey measures. Tables 1 and 2 list selected survey measures that were completed by patients and support persons during baseline and/or follow-up assessments. Included in each table is the measure, concept category, survey instrument and when the measure was assessed.

## Data Availability

The study investigators will make de-identified data sets available for sharing after the trial is finished and primary analyses have been completed and published. Researchers requesting data must present an IRB-approved methodological protocol and explain the relevance of their interest in the study completed data to public health goals. Authors completing secondary analyses of the shared study data must agree to the Center for Clinical Trials and Data Coordination (CCDC) policy on data sharing and publishing. All secondary analysis authors will be expected to credit the primary investigators and mention the data source in all publications. Secondary authors will acknowledge that the data use was in accordance with CCDC protocol and the signed Data Use Agreement (DUA). The University of Pittsburgh Principal Investigator and study coordinator will not release any data until all request criteria are met and a signed Data Use Agreement is filed.

## Abbreviations

AE: Adverse event
CHW: Community health worker
CM: Care management
DSMES: Diabetes self-management education and support
EMR: Electronic medical record
FQHC: Federally Qualified Health Center
FAM-ACT: Family Support for Health Action
GED: General Educational Development
HbA1c: Hemoglobin A1c
I-DSMES: Individual patient-focused diabetes self-management education and support
IRB: Institutional Review Board
ITT: Intention-to-treat
PI: Principal investigator
RA: Research assistant
RCT: Randomized clinical trial
SAE: Serious adverse event
SP: Support person
T2D: Type 2 diabetes
