# Supplementary material for: Comparing the effectiveness of Family Support for Health Action (FAM-ACT) with traditional community health worker-led interventions to improve adult diabetes management and outcomes: study protocol for a randomized controlled trial
Source: Trials. 2022 Oct 3;23:841. doi: 10.1186/s13063-022-06764-1 (PMC9527393; doi:10.1186/s13063-022-06764-1)
Supplement: Supplementary file 1 — Additional file 1. Fidelity Checklists. FAM-ACT Fidelity Checklists. This file contains the checklists used by the PI, co-investigators and CHW manager assessing structured contacts between study staff and participants for fidelity to the protocol. There is one checklist for each type of contact. [file 13063_2022_6764_MOESM1_ESM.pdf]

FAM-ACT Fidelity Form
Recruitment and Screening Calls

FAM-ACT

Did the Research Associate…			
Open with a warm greeting and introduction?	No	Yes	
Speak slowly and use a pleasant tone of voice?	No	Yes	
Read each screening item and its introduction in its entirety?	No	Yes	
Give respondents ample time to answer questions?	No	Yes	
Repeat a screening question **in its entirety** (if asked)?	No	Yes	N/A
Maintain energy and engagement throughout the calls?	No	Yes	
Avoid giving any reaction (stay neutral) or advice in response to participant answers to screening items	No	Yes	

General notes (any rater):

____

____

____

____

____

Areas of competence:

____

____

____

____

____

Areas of growth:

____

____

____

____

____

FAM-ACT Fidelity Form
Informed Consent

Did the Research Associate…			
Read the whole document **OR** review key points in each section with the prospective subject?	No	Yes	
State that the study involves research, the purposes of the research, and how long they will participate in the study?	No	Yes	
Explain how the prospective subject will receive usual care from CHASS Center no matter what program they are part of or if they decide not to participate?	No	Yes	
Explain what is involved in each of the two study arms?	No	Yes	
Explain the risks and the benefits of the study?	No	Yes	
Explain our methods to protect the confidentiality of study data?	No	Yes	
Explain how the prospective subject will be randomized?	No	Yes	
Explain how the prospective subject will be withdrawn from the study?	No	Yes	
Ask if the prospective subject wants more time to read over the consent before deciding whether or not to participate?	No	Yes	
Ask the prospective subject open-ended questions to ensure they are understanding the key information, such as “tell me what you understand about what we will be doing in this study? About the possible harms from this study?	No	Yes	
Give the prospective subject time to ask questions, and answer any questions thoroughly and patiently?	No	Yes	N/A
Verify signatures were obtained, each with a date and time?	No	Yes	
Give or mail (if consent is done virtually) the participant a copy of the fully completed and signed consent document for their records and instruct him/her to retain that copy for reference?	No	Yes	

Additional Notes or Comments (any rater):

____

____

____

____

FAM-ACT Fidelity Form
Survey Assessments

**Did the Research Associate…**			
Speak slowly and use a pleasant tone of voice?	No	Yes	
Read each item and its introduction in its entirety?	No	Yes	
Give respondents ample time to answer questions?	No	Yes	
Thoughtfully redirect the participant if they deviated from the survey?	No	Yes	
Encourage participants to listen to the entire question before accepting the participant' s answer?	No	Yes	
Repeat a question **in its entirety** (if asked)?	No	Yes	N/A
If participant says they do not understand the question, give optional information IF contained in the survey script, otherwise instruct participant to please choose the best possible answer according to their interpretation of the question.	No	Yes	N/A
Repeat **all** of the response options for a question (if asked)	No	Yes	N/A
Probe a "don't know" response at least once if given by the patient? (e.g. please pick the answer closest to your experience, or please answer according to your interpretation of the question)	No	Yes	N/A
Maintain energy and engagement throughout the assessment	No	Yes	
Avoid giving any reaction (stay neutral) or advice in response to participant answers to survey items	No	Yes	

General notes (any rater):

____

____

____

____

____

Areas of competence:

____

____

____

____

____

Areas of growth:

____

____

____

____

____

_

FAM-ACT Fidelity Form
Intro Session

FAM-ACT

Approach

How often did the CHW…				
1. Open with warm greeting and introduction?	Not at all	Somewhat	A lot /consistently	N/A
2. Encourage participants to work together during the session?	Not at all	Somewhat	A lot /consistently	N/A
3. Use open-ended questions?	Not at all	Somewhat	A lot /consistently	N/A
4. Elicit ideas from participants	Not at all	Somewhat	A lot /consistently	N/A
5. Prompt participants to contribute (PT/SP)	Not at all	Somewhat	A lot /consistently	N/A
6. Give participant time to describe their experience?	Not at all	Somewhat	A lot /consistently	N/A
7. Use reflective listening?	Not at all	Somewhat	A lot /consistently	N/A
8. Express empathy and acceptance? (avoid judgmental statements)	Not at all	Somewhat	A lot /consistently	N/A
9. Affirm participant's efforts or commitment?	Not at all	Somewhat	A lot /consistently	N/A

Content

Note: It is expected that CHW will tailor parts of the discussion to the participants' needs, so some subtopics may not be covered

Introduction

- ☐ Covered ground rules
- ☐ Explained how diabetes can cause complications
- ☐ Reviewed patient's diabetes health summary
- ☐ Clearly communicated that patient can do things to prevent complications

PT-SP Teamwork only (not covered in Patient-only intro session)

- ☐ Discussed the importance of weekly talks
- ☐ Discussed how to get the conversation going
- ☐ Reviewed how to stay positive
- ☐ Reviewed Do's and Don'ts for the Support Person to do for the patient

Goal Setting

- ☐ Communicated the importance of choosing a goal that's important to the patient
- ☐ Discussed main components of I-SMART action planning

Wrap-Up

- ☐ Encouraged PT/SP to look at the DSMES class schedule and pick a date to attend
- ☐ Reviewed the I-SMART action plan

General notes (any rater):

____

____

____

____

Important context (for example, setting, distractions, patient context) that affected session (any rater):

____

____

Topics added and reasons for that (any rater):

____

Reasons for topics omitted, if any (any rater):

____

Areas of competence:

____

____

____

____

____

Areas of Growth:

____

____

____

____

____

FAM-ACT Fidelity Form
Diabetes Education Sessions

FAM-ACT

Session Number: ________

Approach

How effectively did the CHW…	Not at all	Somewhat	A lot/ consistently	N/A
Convey energy				
Track progress through agenda and manage the pace				
Create a positive atmosphere (avoid pessimism, put downs)				
Encourage participation (round robin, open ended Qs, redirect Qs to other participants)				
Convey that participants are empowered to make change				
Show active listening including reflective statements				
Give frequent affirmations (e.g. for anything positive such as trying to learn something or make a change)				
Manage conflict between participants skillfully				
Manage difficult participants skillfully (talking too much, rude, know-it-all, etc.)				

Education Topics

Instructions: Session topics are covered in both the Conversation maps and Support Person- focused sessions. Put a checkmark in the box if the topic was covered. Some recordings will not contain Support Person-focused sessions.

Conversation Map and other included Topics	DSMES Class	Add-On Session/ Discussed SP Role
Increasing physical activity	☐	☐
Uplifting mood	☐	☐
Stress management	☐	☐
Complications	☐	☐
Healthy eating	☐	☐
Meal prep & planning meals	☐	☐
Grocery shopping	☐	☐
Reading nutrition labels	☐	☐
Healthy eating topics	☐	☐
Challenging food situations	☐	☐
High sugar symptoms	☐	☐
Low sugar symptoms	☐	☐
Sick days	☐	☐
Diabetes screening tests	☐	☐
Foot care	☐	☐
Health emergencies	☐	☐
Medication adherence	☐	☐
Healthcare provider visits	☐	☐

General feedback (any rater):

Feedback on discussion of SP's role in helping patient with their diabetes management [SP-focused intervention only]

Important context (for example, setting, distractions, patient context) that affected session:

Topics added and/omitted and reasons for that (any rater):

Areas of competence:

Areas of growth:
