# Supplementary material for: Comparing the effectiveness of Family Support for Health Action (FAM-ACT) with traditional community health worker-led interventions to improve adult diabetes management and outcomes: study protocol for a randomized controlled trial
Source: Trials. 2022 Oct 3;23:841. doi: 10.1186/s13063-022-06764-1 (PMC9527393; doi:10.1186/s13063-022-06764-1)
Supplement: Supplementary file 2 — Additional file 2. Fidelity-Monitoring-Schedule. Intervention component fidelity monitoring schedule. The table located in the file displays the fidelity monitoring schedules by study contact, staff member and participant type. [file 13063_2022_6764_MOESM2_ESM.docx]

**Additional File 1.** Intervention component fidelity monitoring schedule

The table displays fidelity monitoring schedules by type of study contact. The first column lists components of the study protocol that involve direct interaction of a study staff member (second column) and either a patient or a SP (third column). As can be seen, monitoring will take place more frequently during the first year the study is active and less frequently thereafter. If necessary, fidelity monitoring may be conducted more frequently than described in the monitoring. For example, contacts involving a new staff member who joins the study after the first year may need to be monitored more frequently than the rest of the staff.

|  |  |  | **Monitoring Schedule** | |
| --- | --- | --- | --- | --- |
| **Study contact** | **Staff member** | **Participant(s)** | **First year** | **Subsequent years** |
| Recruitment calls | RA | Patient | 2 calls per RA | 1 call per RA per year |
| Informed consent and baseline assessments | RA | Patient, SP | 2 patients and 2 SPs per RA | 1 patient and 1 SP per RA per year |
| Intro sessions | CHW | Patient, SP | 4 support-enhanced sessions per CHW | 1 support-enhanced session per CHW every 6 months |
| DSME group sessions and support-enhanced extenders | CHW | Patient, SP | 1 of each session (1-6) per CHW within the first 6 months | Randomly selected 1 session per CHW every 6 months |
| 6-Month Assessments | RA | Patient, SP | 2 patients and 2 SPs per RA | 1 patient and 1 SP per RA per year |
| 12-Month Assessments | RA | Patient | 2 patients per RA | 1 patient per RA per year |

CHW, Community Health Worker; DSME, diabetes self-management education; RA, research assistant
