# Supplementary material for: Comparing the effectiveness of Family Support for Health Action (FAM-ACT) with traditional community health worker-led interventions to improve adult diabetes management and outcomes: study protocol for a randomized controlled trial
Source: Trials. 2022 Oct 3;23:841. doi: 10.1186/s13063-022-06764-1 (PMC9527393; doi:10.1186/s13063-022-06764-1)
Supplement: Supplementary file 3 — Additional file 3. Additional-Patient-&-SP-Measures. Table 1, Select patient survey measures; Table 2, Select support person survey measures. Tables 1 and 2 list selected survey measures that were completed by patients and support persons during baseline and/or follow-up assessments. Included in each table is the measure, concept category, survey instrument and when the measure was assessed. [file 13063_2022_6764_MOESM3_ESM.docx]

**Supplementary Table 1.** Select patient survey measures

| **Measure** | **Concept Category** | **Instrument** | **Baseline** | **6-Months** | **12-Months^a^** |
| --- | --- | --- | --- | --- | --- |
| Depressive and anxiety symptoms | Medical/mental health general | Patient Health Questionnaire (PHQ-4)^1^ | X | X |  |
| Self-rated health status | Medical/mental health general | Self-rated health question (SF-1)^2^ | X | X |  |
| Functional status | Medical/mental health general | PROMIS Physical Function Short Form 4a^3^ | X | X |  |
| **Diabetes Distress** | Psychological behavior determinants | Problem Areas in Diabetes (PAID) - 5 for People with Diabetes^4^ | X | X | X |
| **Patient Activation** | Psychological behavior determinants | Patient Activation Measure -10 (PAM)^5^ | X | X | X |
| **Diabetes self-efficacy** | Psychological behavior determinants | Self-Efficacy for Managing Chronic Diseases Scale^6,7^ | X | X | X |
| Goal setting behavior | Psychological behavior determinants | Adapted from Patient Assessment of Chronic Illness Care (PACIC) Goal setting subscale^8^ | X | X | X |
| Patient Activation in Medical Visits | Patient engagement in healthcare | Perceived Efficacy in Patient-Physician Interactions scale (PEPPI)^9,10^ | X | X | X |
| Health literacy | Education/literacy | Health Literacy Screening Questions^11^ | X |  |  |
| Importance of diabetes | Competing factors | Single item: I have more important things in my life to take care of than diabetes to take care of now. (5-point scale, 1-strongly disagree to 5-strongly agree)^12,13^ | X | X |  |
| Diabetes self-care behaviors **– healthy eating, physical activity,** blood sugar testing, foot care | Self-management behaviors | Summary of Diabetes Self-Care Activities Measure (SDSCA)^14^ | X | X | X |
| **Medication adherence** | Self-management behaviors | Summary of Diabetes Self-Care Activities Measure (SDSCA) | X | X | X |
| Smoking - cut down/quit attempt | Health behavior | Behavioral Risk Factor Surveillance System (BRFSS)^15^ |  | X | X |
| Smoking status | Health behavior | Global Adult Tobacco Survey (GATS)^16^ | X | X | X |
| Smoking status (# cigarettes per day) | Health behavior | Global Adult Tobacco Survey (GATS) | X | X | X |
| Patient's General Perceived Social Support | Social support general | ENRICHD Social Support Instrument (ESSI)^17^ | X | X |  |
| **Patient overall satisfaction with SP support for diabetes** | Patient perception of SP help | Patient overall satisfaction with SP support items created for CO-IMPACT Study^18,b^ | X | X | X |
| **Patient perceived supportive vs. non-supportive SP behaviors** | Patient perception of SP help |  | X | X | X |
| Autonomy support (“supportive behaviors”) |  | Important Other Climate Questionnaire (IOCQ)^19^ |  |  |  |
| Non-supportive behaviors |  | 3 items created for this study^c^ |  |  |  |
| SP Helps with Medical Visits | SP roles, behaviors, and attitudes | Items created for CO-IMPACT Study^18^ | X | X | X |
| SP Roles in Patient Diabetes Management Care | SP roles, behaviors, and attitudes | Items created for CO-IMPACT Study^18,d^ | X | X | X |
| **Impact of COVID on ability to manage diabetes** | COVID-19 impact | Single item created for study^e^ |  | X | X |

**Bold typeface**, change in measure from baseline to 6-months will be examined as a secondary outcome; ENRICHD Enhancing Recovery in Coronary Heart Disease Study; CO-IMPACT; Caring Others Increasing Engagement in Patient Aligned Care Teams Study; SP, support person

^a^Per the COVID-adapted protocol, 12-month assessments will be completed only if study timeline permits.

^b^Two items assessing patient’s satisfaction with the support they receive from their SP and whether they feel like they would be worse off without their SP’s help with their diabetes care

^c^Non-supportive behaviors will be assessed with 3 items structured similarly to the IOCQ items addressing SP irritation, criticism and argumentativeness.

^d^Example roles include suggesting questions prior to patients’ health care appointments, accompanying the patient in the exam room, discussing the visit after it has ended

^e^“In the last six months, how have the COVID pandemic or social distancing rules affected your ability to manage your diabetes?” (5-point scale, 1-much harder to 5-much easier)

**Supplementary Table 2.** Select support person survey measures

| **Measure** | **Concept Category** | **Instrument** | **Baseline** | **6-Months** |
| --- | --- | --- | --- | --- |
| Importance of diabetes | Competing factors | Single item: I have more important things in my life to take care of than helping <Patient> with their health care(5-point scale, 1-strongly disagree to 5-strongly agree)^12,13^ | X | X |
| **Self-efficacy for helping patient with diabetes** | Health behavior determinant | Self-Efficacy for Managing Chronic Diseases Scale (adapted for support person)^6,7^ | X | X |
| Goal setting (help patient set goal) | SP roles, behaviors, and attitudes | Adapted from Patient Assessment of Chronic Illness Care (PACIC) Goal setting subscale^8^ | X | X |
| Supporter perception of patient empowerment | SP roles, behaviors, and attitudes | DAWN Family Experience of Patient Involvement scale (DFEPI)^20^ | X | X |
| **Diabetes distress** | SP roles, behaviors, and attitudes | Problem Areas in Diabetes (PAID) - 5 for Family Members^4^ | X | X |
| Caregiving burden | SP burden | DAWN Impact of Diabetes Profile - Family Members (DIDP-FM) plus additional question^20,a^ | X | X |

**Bold typeface**, measure will be examined as a secondary outcome

^a^In the last 6 months, how much of a burden has it been for you to help [Patient] manage their diabetes? (5-point scale: 1-very large burden to 5-no burden)

**Table References**

1. Kroenke K, Spitzer R, Williams J, Lowe B. An ultra-brief screening scale for anxiety and depression: The PHQ–4. *Psychosomatics*. 2009;50(6):613-621.

2. Ware JE. *How to Score and Interpret Single-Item Health Status Measures: A Manual for Users of the of the SF-8 Health Survey:(With a Supplement on the SF-6 Health Survey)*. QualityMetric, Incorporated; 2001.

3. Dewitt B, Feeny D, Fischhoff B, et al. Estimation of a preference-based summary score for the Patient-Reported Outcomes Measurement Information System: The PROMIS®-Preference (PROPr) Scoring System. *Med Decis Making*. 2018;38(6):683-698. doi:10.1177/0272989X18776637

4. McGuire BE, Morrison TG, Hermanns N, et al. Short-form measures of diabetes-related emotional distress: the Problem Areas in Diabetes Scale (PAID)-5 and PAID-1. *Diabetologia*. 2010;53(1):66-69. doi:10.1007/s00125-009-1559-5

5. Hibbard JH, Stockard J, Mahoney ER, Tusler M. Development of the Patient Activation Measure (PAM): conceptualizing and measuring activation in patients and consumers. *Health Serv Res*. 2004;39(4 Pt 1):1005-1026. doi:10.1111/j.1475-6773.2004.00269.x

6. Lorig K, Stewart A, Ritter P, Gonazalez V, Laurent D, Lynch J. *Outcomes Measures for Health Education and Other Health Care Interventions.* Sage Publications; 1996.

7. Bodenheimer T, Lorig K, Holman H, Grumbach K. Patient self-management of chronic disease in primary care. *JAMA*. 2002;288(19):2469-2475. doi:10.1001/jama.288.19.2469

8. Glasgow RE, Wagner EH, Schaefer J, Mahoney LD, Reid RJ, Greene SM. Development and validation of the Patient Assessment of Chronic Illness Care (PACIC). *Med Care*. 2005;43(5):436-444. doi:10.1097/01.mlr.0000160375.47920.8c

9. Maly RC, Frank JC, Marshall GN, Robin M, Reuben DB. Perceived Efficacy in Patient–Physician Interactions (PEPPI): Validation of an instrument in older persons. *Journal of the American Geriatrics Society*. 1998;46(7):889-894.

10. ten Klooster PM, Oostveen JC, Zandbelt LC, et al. Further validation of the 5-item Perceived Efficacy in Patient-Physician Interactions (PEPPI-5) scale in patients with osteoarthritis. *Patient Education and Counseling*. 2012;87:125-130. doi:10.1016/j.pec.2011.07.017

11. Chew LD, Bradley KA, Boyko EJ. Brief questions to identify patients with inadequate health literacy. *Family Medicine*. 2004;36(8):588-594.

12. Kerr EA, Heisler M, Krein SL, et al. Beyond comorbidity counts: how do comorbidity type and severity influence diabetes patients’ treatment priorities and self-management?[see comment]. *Journal of General Internal Medicine*. 2007;22(12):1635-1640.

13. Zulman DM, Rosland AM, Choi H, Langa KM, Heisler M. The influence of diabetes psychosocial attributes and self-management practices on change in diabetes status. *Patient Educ Couns*. 2012;87(1):74-80. doi:10.1016/j.pec.2011.07.013

14. Toobert DJ, Hampson SE, Glasgow RE. The summary of diabetes self-care activities measure: results from 7 studies and a revised scale. *Diabetes Care*. 2000;23(7):943-950.

15. *Behavioral Risk Factor Surveillance System Survey Questionnaire*. Centers for Disease Control and Prevention (CDC); 2003.

16. Global Adult Tobacco Survey Collaborative Group. Tobacco Questions for Surveys: A Subset of Key Questions from the Global Adult Tobacco Survey (GATS). Published online 2011.

17. Mitchell PH, Powell L, Blumenthal J, et al. A Short Social Support Measure for Patients Recovering From Myocardial Infarction: THE ENRICHD SOCIAL SUPPORT INVENTORY. *Journal of Cardiopulmonary Rehabilitation*. 2003;23(6):398-403. doi:10.1097/00008483-200311000-00001

18. Rosland AM, Piette JD, Trivedi R, et al. Effectiveness of a Health Coaching Intervention for Patient-Family Dyads to Improve Outcomes among Adults with Diabetes: A Randomized Clinical Trial. *JAMA Network Open*.

19. Williams G, Lynch M, Ryan R, Sharp D, Deci E. Validation of the important other climate questionnaire: Assessing autonomy support for health-related change. *Families, Systems, & Health*. 2006;24(2):179-194.

20. Kovacs Burns K, Nicolucci A, Holt RIG, et al. Diabetes Attitudes, Wishes and Needs second study (DAWN2^TM^): Cross-national benchmarking indicators for family members living with people with diabetes. *Diabet Med*. 2013;30(7):778-788. doi:10.1111/dme.12239
